# Effects of a plank-based strength training programme on muscle activation in patients with long COVID: a case series

**DOI:** 10.23938/ASSN.1083

**Published:** 2024-10-02

**Authors:** Sofía Laguarta-Val, María Carratalá-Tejada, Francisco Molina-Rueda, Ricardo Moreta-Fuentes, Diego Fernández-Vázquez, Raúl López-González, Carmen Jiménez-Antona, César Moreta-Fuentes, Alberto Javier Fidalgo-Herrera, Juan Carlos Miangolarra-Page, Víctor Navarro-López

**Affiliations:** 1 Rey Juan Carlos University Faculty of Health Sciences Physical Therapy, Occupational Therapy, Rehabilitation and Physical Medicine Department Madrid Spain; 2 Rey Juan Carlos University Faculty of Health Sciences Movement Analysis, Biomechanics, Ergonomics, and Motor Control Laboratory Madrid Spain; 3 Fisyos Center Madrid Spain; 4 FREMAP Care Center Fuenlabrada (Madrid) Spain; 5 Alfonso X el Sabio University Villanueva de la Cañada (Madrid) Spain; 6 University Hospital of Fuenlabrada Physical Medicine and Rehabilitation Madrid Spain

**Keywords:** COVID-19, Exercise Therapy, Rehabilitation, Muscle Strength, COVID-19, Terapia por Ejercicio, Rehabilitación, Fuerza muscular

## Abstract

**Background:**

This study aimed to analyse the effects of a plank-based strength training programme on muscle activation in patients with long COVID.

**Subjects and methods:**

Case series study that included patients with long COVID who participated in a 12-week trunk and pelvic muscle strength training programme. Clinical variables and the modified fatigue impact scale (MFIS) were used to assess fatigue levels. Percentage of muscle activation during a core muscle plank was measured via surface electromyography. Pre- and post-intervention results were compared using the Wilcoxon signed-rank test and evaluated with Cohen’s D effect size (ES).

**Results:**

Twenty-one subjects participated in the study; 81% female, mean age 47.5 years (range: 28-55 years), and median duration of symptoms 21 months (range: 5-24 months); 90.5% of the participants experienced fatigue (MFIS score ≥ 38). Muscle activation during plank exercises improved across all muscles after the intervention, with significant increases in the left (p = 0.011, medium ES) and right external oblique (p =0.039, small ES) muscles and the right latissimus dorsi muscle (p = 0.039, small ES). Additionally, significant reductions in fatigue were observed in the total MFIS score (p = 0.004, medium ES) and in the physical (p < 0.001, large ES) and psychosocial subscales (p = 0.033, small ES).

**Conclusions:**

Results suggest that a plank-based strength training programme may be effective in enhancing trunk and pelvic muscle activation in individuals with long COVID.

## RESUMEN

**Fundamento:**

Analizar los efectos de un programa de fuerza basado en ejercicios de planchas abdominales sobre la activación muscular en pacientes con COVID persistente (CP).

**Sujetos y métodos:**

Serie de casos de pacientes con CP que realizaron un programa de entrenamiento de fuerza de los músculos del tronco y la pelvis durante doce semanas. Se midió la fatiga mediante la Escala de Impacto de Fatiga Modificada (MFIS) y se recogieron variables clínicas. El porcentaje de activación muscular durante una plancha de músculos centrales se midió mediante electromiografía de superficie, se comparó antes y después de la intervención mediante Wilcoxon y se evaluó el tamaño del efecto (TE) mediante la d de Cohen.

**Resultados:**

Participaron 21 sujetos, 81% mujeres, con edad media 47,5 años (rango: 28-55), mediana del tiempo con síntomas 21 meses (rango: 5-24) y 90,5% con fatiga (puntuación MFIS ≥38). El porcentaje de activación muscular durante el ejercicio de plancha mejoró después de la intervención para todos los músculos, pero fue significativamente mayor para el oblicuo externo izquierdo (p=0,011, TE medio) y derecho (p=0,039, TE pequeño) y para el dorsal ancho derecho (p=0,039, TE pequeño). Se observó una reducción significativa de la fatiga en la puntuación total de MFIS (p=0,004, TE medio) y en las subescalas física (p<0,001, TE grande) y psicosocial (p=0,033, TE pequeño).

**Conclusiones:**

Los resultados sugieren que el programa de entrenamiento de fuerza basado en ejercicios de plancha podría ser beneficioso para mejorar la activación de los músculos de tronco y pelvis en personas con CP.

## INTRODUCTION

Long COVID (LC) is a heterogeneous condition described as an elapsed, acute infectious period^1^. The World Health Organization defines it as follows: *Post COVID-19 condition occurs in individuals with a history of probable or confirmed SARS-CoV-2 infection, usually 3 months from the onset of the COVID-19 with symptoms and that last for at least 2 months and cannot be explained by an alternative diagnosis*. Several common symptoms can affect daily functioning, including fatigue, shortness of breath, and cognitive issues, among others. The symptoms may emerge after the initial recovery from acute COVID-19 or persist from the original illness. Moreover, symptoms can fluctuate or recur over time^2^.

The most commonly reported symptoms are fatigue (53%), dyspnoea (43.4%), joint pain (27.3%), and chest pain (21.7%)^3^^,^^4^. Several studies have reported that chronic fatigue is the most prevalent symptom of LC^5^^,^^6^. Regarding musculoskeletal impairment, the most prevalent muscle manifestations of LC include myalgia, muscle weakness, decreased muscular strength, and a decline in physical performance^7^. Contributing factors to persistent musculoskeletal symptoms may include muscle cell damage, the hyper-inflammatory state induced by COVID-19 infection, hypoxemia, mitochondrial damage, and dysregulation of the renin-angiotensin system^8^.

The prevention and management of LC remain inadequate, as many aspects of the condition still require further clarification^7^. Evidence suggests that exercise may help improve symptoms in patients with LC^7^; however, the role of physical activity has not been fully clarified^9^ and the effectiveness of exercise interventions is still under investigation^10^. Several studies have focused on cardiopulmonary rehabilitation^11^^,^^12^, aerobic and resistance training^13^, or respiratory physiotherapy^14^. To the best of our knowledge, there are no studies analysing the impact of strengthening exercise programs in patients with LC.

Extensive weakness of the muscles of the lower limbs and trunk have been described following COVID-19 infection. Paneroni et al. demonstrated a decrease in quadriceps muscle strength in hospitalized patients diagnosed with severe COVID-19, as measured by functional tests such as the maximal voluntary contraction test^15^. Studies involving skeletal muscle biopsies (quadriceps *femoris*, *tibi-alis* anterior, and psoas or *rectus abdominis*) have revealed the existence of myopathies, myositis, and muscle atrophy^16^. This weakness may compromise the performance of daily living activities^7^.

A stable, upright posture was achieved through the synergy between limb and trunk muscles^17^, making core stability, defined as the ability to control the trunk during static and dynamic tasks, one of the main elements of balancing. Core exercises are commonly used in rehabilitation to enhance trunk stability, and plank exercises are effective for strengthening the trunk (*rectus abdominis*^18^, obliques^19^^,^^20^, and *latissimus dorsi*^21^), pelvis (gluteal musculature^22^), and lower limb muscles (*rectus fe-moris*^21^). Additionally, the perceived exertion during the plank exercises is lower compared to other dynamic exercises^21^. The hypothesis of this study is that a core-strengthening exercise intervention in patients with LC will improve abdominal activation.

The purpose of this study was to analyse the effects of a plank-based strength training programme on trunk and pelvic muscle activation in patients with LC.

## MATERIALS AND METHODS

*Design.* This case series study was conducted at the Movement Analysis, Biomechanics, Ergonomics, and Motor Control Laboratory (*LAMBECOM*, *Laboratorio de Análisis del movimiento, Biomecánica, Ergonomía y Control motor*) within the Faculty of Health Sciences at Rey Juan Carlos University in Madrid, Spain, between January and May 2022. Study participants were diagnosed with LC and the impact of a plank-based core-stabilization program on muscle activation and fatigue was assessed.

*Participants.* Study participants were selected by consecutive, non-probability sampling from *Asociación Madrileña de COVID Persistente* (*AMACOP*, Long COVID Association, Madrid, Spain). Inclusion criteria: 1) LC symptoms for more than a year; 2) aged 18 years or older; and 3) complete vaccination schedule for COVID-19, according to the criteria of the Spanish Ministry of Health. Patients were excluded if they had any of the following conditions: severe heart disease, abdominal hernia, a history of musculoskeletal injuries or surgery in either lower or upper limbs in the last year, the coexistence of neuromuscular pathology. Pregnant females were also excluded from the study.

*Ethical aspects.* Written informed consent was obtained before any procedure was initiated. The study was carried out in accordance with the principles of the Declaration of Helsinki and the Biomedical Research Act 14/2007. This study has been reviewed and approved (reference number 21/173) by the Ethics Committee of *Hospital Universitario Fundación Alcorcón*.

*Procedures.* No substantial changes were made to the methods after the study commenced. Screening began in January 2022 and the last patient was evaluated in May 2022.

All participants underwent a clinical examination at *LAMBECOM* and the following physical and clinical characteristics were collected: sex (male, female), age (years), weight (kg), height (cm), body mass index (BMI, kg/m^2^), duration of symptoms (months), hospitalization due to COVID (yes, no), re-infections (yes, no), and other pathologies (yes, no).

Next, each participant was instructed to perform a high plank for 40 seconds. The exercise was repeated three times, with a 1-minute rest between repetitions. A high plank is a bodyweight exercise performed by getting on all fours and placing the hands directly under the shoulders. The legs are straightened to lift the knees off of the floor. Muscle activity was measured bilaterally using surface electromyography (sEMG) during the high plank of the rectus abdominis (RA), external oblique (EO), internal oblique (IO), *gluteus maximus* (GMAX), *gluteus medius* (GMED), *latissimus dorsi* (LD), and *erector spinae* (ES). Electromyographic evaluations were carried out using the Cometta Zero^®^ wireless EMG system (Milan, Italy). The skin was shaved and cleaned with alcohol. For each muscle, two adhesive Ag/AgCl electrodes were placed according to the specifications of SENIAM^28^ and Choi et al. 2021^29^. A manual muscle test was performed to verify the correct positioning of the electrodes. sEMG signals were acquired at a sampling frequency of 1,000 Hz. The recordings were processed to remove noise. sEMG signals were filtered using a 20-Hz high-pass second-order filter and a 450-Hz low-pass fourth-order filter. The linear envelope was obtained using a root mean square (RMS) moving average with a 100 ms window and an overlap of 10 ms. The mean amplitude of the RMS sEMG signals was calculated over the full 40-second duration of each plank. sEMG amplitude, frequency, and duration are affected by various factors such as muscle fibre type, speed of the actions, subcutaneous fat thickness, and electrode placement. To minimize inter-subject and inter-experiment variability, amplitude normalization was performed based on the maximum RMS peak during the plank for each individual and muscle separately. Normalizing to the peak or mean amplitude during the activity of interest has been shown to reduce variability between individuals more effectively than using raw EMG data or normalizing to maximum voluntary isometric contractions^30^.

To assess the level of physical activity of the participants, the International Physical Activity Questionnaire (IPAQ) short form was used. This tool has demonstrated acceptable validity for assessing physical activity levels and patterns in healthy adults^23^. It consists of seven items and gathers information on the time spent in moderate- and vigorous-intensity activities, walking, and sitting. It evaluates the intensity (light, moderate, or vigorous), frequency (days per week), and duration (time per day) of physical activity. Participants are categorized into three activity levels based on their metabolic equivalent of task (MET) consumption: Category 1, low, Category 2, moderate, and Category 3, high^24^^,^^25^.

To evaluate the participants’ fatigue before and after the intervention, the Modified Fatigue Impact Scale (MFIS) was used. This widely used tool is a shorter version of the Fatigue Impact Scale, comprising 21 items compared to the original 40^26^. It assesses perceived fatigue over the past four weeks, providing information on overall fatigue as well as physical, cognitive, and psychosocial sub-scales. The total score ranges from 0 to 84, with 84 indicating the maximum fatigue. Most studies use a score of 38 as the cut-off point^27^.

*Intervention.* Participants engaged in a plank-based core-stabilization program designed to train muscles and neuromuscular control^31^. These exercises are intended to enhance power, strength, balance, and proprioception^17^. The programme was carried out following to MORETA methodology^®^ and was supervised by two physiotherapists during all sessions. For improved instruction, participants were divided into two groups.

The core-stabilization program consisted of 60-minute sessions conducted twice a week for 12 weeks. Each session included a 10-minute warm-up (stretching exercises), followed by 40 minutes of core training involving abdominal and plank exercises without external weights (Table 1), and concluded with a 10-minute cool-down (breathing, stretching, and muscle relaxation).

**Table 1:** Core training during the 12-weeks intervention

Exercise	Sets	Repetitions per set	Observations
Sit-ups	3	30-60
Toe Bridge Prone (High Plank with elbows extended or Straight Arm Plank and Forearm Plank	3	4	Held position for 15-30 s, up to 60 s
Pelvic Bridge	1	10	Held position for 60 s
Lateral bridge	3	4 on each side	Held position for 15-30 s, up to 60 s
Crunch	3	20-30	

During the training, patients were instructed to contract their abdominal muscles maximally for as long as possible while maintaining correct posture. Rest periods between repetitions included one minute of passive recovery. The intensity of the exercises was maintained at a somewhat-hard level^13^ according to the Rating of Perceived Exertion Category Scale^32^.

*Statistical analysis*. Statistical analyses were conducted using the SPSS software (version 27.0; IBM Corp, Armonk, NY). The Shapiro-Wilk test was performed, revealing that the distribution of all variables did not follow a normal distribution (p-value < 0.05); thus, non-parametric tests were used. Descriptive statistics (percentages, median, interquartile range, maximum, and minimum) were calculated. An α level of 0.05 and 95% confidence intervals (CI) were used in all analyses. The Wilcoxon test was used to compare the mean range of related samples (pre-post). Statistical significance was set at p < 0.05. Cohen’s D was used to estimate the effect size; an effect size of D ≥ 0.2 was considered small, D ≥ 0.5 medium, and D ≥ 0.8 large.

## RESULTS

A total of 21 subjects participated in this case series study. Of these, 81% were female; mean age was 47.5 years (range: 28-55 years); 76.2% of the participants were at least moderately active and half had other pathologies (Table 2). The median duration of symptoms was 21 months (with a minimum of five months and a maximum of 19 months). Nearly 29% of participants had required hospitalization and almost all experienced fatigue according to the MFIS. Only two out of the 21 subjects (9.52%) scored below 38 points on the MFIS scale (with scores of 32 and 15 points, indicating no fatigue); these individuals were 52 and 42 years old, with symptoms present 24 and 17 months, weighed 62.5 kg and 85.8 kg, and measured 172 and 175 cm, respectively.

**Table 2:** Clinical characteristics of participants

Variable	Median	Q1-Q3
Age (years)	47.5	28-55
Weight (Kg)	67.0	58.35-81.35
Height (cm)	165.0	161-169
BMI (Kg/m^2^)	26.8	21.67-29.3
Time with symptoms (months)	21.0	14.5-23
*Variable*	*n*	*%*
Sex (female)	17	81
Level of physical activity (IPAQ)		
Low	5	23.8
Moderate	11	52.4
Vigorous	5	23.8
Fatigue (MFIS) (yes)	19	90.5
Hospitalization (yes)	6	28.6
Re-infections (yes)	3	14.3
Other pathologies (yes)	12	57.1

BMI: body mass index; IPAQ: International Physical Activity Questionnaire; MFIS: Modified Fatigue Impact Scale.

Significant differences in muscle activation percentages during planks were observed for the EO muscle (higher post-intervention, with an effect size medium for the left EO and small for the right EO) and for the right LD (higher post-intervention with a small effect size). Muscle activation of the remaining muscles improved post-intervention, but these changes were not statistically significant (Table 3).

**Table 3:** Muscle activity percentages recorded using surface electromyography

Muscle	Pre-Intervention		Post-Intervention		p Wilcoxon	Effect size Cohen’s d
	Median	Q1 - Q3	Median	Q1 - Q3		
*Rectus abdominis*						
left	49.70	42.57 - 54.47	50.96	42.39 - 56.34	0.986	0.003
right	49.66	41.49 - 52.95	51.00	49.37 - 57.76	0.106	0.353
*External oblique*						
left	53.29	45.60 - 56.59	57.06	50.07 - 57.72	0.011	0.558
right	53.74	45.23 - 60.29	57.92	53.17 - 61.19	0.039	0.451
*Internal oblique*						
left	49.47	42.95 - 56.36	53.73	43.28 - 56.91	0.768	0.064
right	52.58	36.83 - 53.86	56.27	45.26 - 57.66	0.073	0.391
*Gluteus maximus*						
left	50.93	20.67 - 69.70	51.76	40.75 - 76.23	0.689	0.087
right	51.12	33.27 - 66.33	48.96	34.29 - 61.49	0.639	0.102
*Gluteus medius*						
left	53.12	38.96 - 57.12	56.16	41.17 - 61.40	0.664	0.095
right	47.62	39.34 - 54.64	56.53	48.33 - 61.65	0.085	0.376
*Latissimus dorsi*						
left	58.76	49.14 - 63.77	58.93	37.35 - 60.50	0.903	0.027
right	53.29	33.76 - 57.14	55.45	48.31 - 64.46	0.039	0.451
*Erector spinae*						
left	51.76	36.58 - 64.69	59.09	44.79 - 65.70	0.339	0.209
right	56.65	34.44 - 61.49	53.85	43.88 - 61.78	0.090	0.027

**Table 4:** Modified Fatigue Impact Scale (MFIS) scoring

MFIS	Pre-Intervention		Post-Intervention		p	Effect size Cohen’s d
	Median	Q1 - Q3	Median	Q1 - Q3		
Total	65	60.0 - 71.5	48	42.0 - 63.5	0.004	0.622
*Subscales*						
Physical	30	24.0 - 33.0	20	16.0 - 28.0	<0.001	0.857
Cognitive	31	26.0 - 34.0	26	21.5 - 33.5	0.250	0.251
Psychosocial	6	3.5 - 7.5	4	2.5 - 6.0	0.033	0.467

In addition, the total MFIS score improved significantly post- intervention, with a medium effect size. The physical and psychosocial subscales also showed significant improvements, with large and small effect sizes, respectively. However, the cognitive subscale improved without reaching statistical significance (Table 4). The two subjects who showed no fatigue on the MFIS scale before the intervention (32 and 15 points) had scores of 28 and 13 points after the intervention.

## DISCUSSION

In the present study, we evaluate the effects of a plank-based strength training program on muscle activation in LC patients who had experienced symptoms for more than three months. No adverse effects were observed during the intervention. There were no attrition rates during treatment, and full adherence was achieved, with all 21 subjects completing the intervention.

Exercise capacity is reduced in individuals susceptible to LC compared to those without symptoms for more than three months following SARS-CoV-2 infection^33^^,^^34^. Therefore, implementing an exercise program focused on physical activity in LC patients is crucial for restoring the physical capacity they had before the infection. To the best of our knowledge, few studies analyse strength training protocols in individuals diagnosed with LC. Kaczmarczyk et al. investigated the effects of an exercise program on muscle weakness in post-COVID survivors. Each training session aimed to achieve an exercise intensity of 70% of one repetition maximum (1RM) and consisted of three sets of 12 repetitions of each exercise (incline bench press, 45-degree leg press, latissimus pull-down, trunk crunch, T-bar row, leg extension, and leg curl). The authors demonstrated that this protocol improved isometric and isokinetic strength in post-COVID survivors^35^.

Muscle weakness, impaired physical activity, and fatigue are three common symptoms experienced by individuals with LC^8^. The training program proposed in our study was designed to enhance the activation of trunk and pelvic muscles. Contraction of the trunk muscles stabilizes the spine and provides improved stabilization for both lower and upper limb movements^17^. Therefore, proximal stabilization of the trunk muscles is essential for performing functional tasks and preventing excessive strain on the spine during movements. In addition, effective activation of the trunk and pelvic muscles is correlated with better balance and functional performance^36^.

After the intervention, improvement of muscle activation of the EO and LD muscles is observed in individuals with LC. In addition, the activation of the gluteus medius muscle improved post-intervention, though the changes did not reach statistical significance. Therefore, while the proposed training protocol may have a functional impact on LC patients, further studies with more rigorous designs are needed to confirm these findings.

As for fatigue, patients show significant improvement in their scores after the three-month program compared to baseline values. Persistent fatigue lasting 16-20 weeks after symptom onset affects 13-33% of individuals with COVID-19^37^. Tabacof et al. demonstrated that the most common persistent symptom reported by LC patients was fatigue (82%)^38^. The impact of persistent fatigue on functionality in individuals with LC has been evaluated, revealing that 15-40% of patients are unable to return to work for 2-4 months after symptom onset^39^^-^^41^. Given the significance and prevalence of fatigue in individuals with LC, it is important to emphasize the need for exercise programs that help reduce fatigue levels (assessed in this study using the MFIS) and potentially improve functionality indirectly. It has also been observed that moderate-intensity aerobic exercise programs can reduce the severity and progression of COVID-19-associated disorders, improve quality of life, alleviate disease symptoms, and have beneficial effects on the immune system^42^^,^^43^.

This study provides practical implications for therapists who design training programs for patients with LC. The core-stability program based on plank exercises can be adapted to individuals with different levels of disability; however, there is limited knowledge about exercise classification for patients with LC. Future studies should explore different exercise modalities and different intensities using randomized controlled trials.

This study has several limitations: 1) due to recruitment difficulties, no control group was included; 2) there was no long-term follow-up to assess the maintenance of the training protocol´s beneficial effects; 3) there was heterogeneity in patient activity levels, fatigue, gender distribution, hospitalization, and reinfection rates; and 4) no functional measures nor objective measures of muscle strength were carried out. The absence of a control group precludes the ability to compare the observed improvements and makes it impossible to rule out the influence of factors other than the intervention itself. The long-term sustainability of the training protocol’s effects cannot be guaranteed due to the lack of follow-up. Given the heterogeneity of the sample, the results should not be generalized.

In conclusion, a plank-based strengthening exercise intervention for LC individuals may improve trunk and pelvic muscle activation and positively affect fatigue perception. The training protocol is well tolerated by LC patients, with no reported adverse effects. However, randomized controlled trials are needed to confirm these findings in LC individuals.
